# Khat and neurobehavioral functions: A systematic review

**DOI:** 10.1371/journal.pone.0252900

**Published:** 2021-06-10

**Authors:** Ayan Ahmed, Manuel J. Ruiz, Kathrin Cohen Kadosh, Robert Patton, Davinia M. Resurrección

**Affiliations:** 1 Faculty of Health and Medical Sciences, School of Psychology, University of Surrey, Guildford, Surrey, United Kingdom; 2 Department of Psychology, University of Extremadura, Badajoz, Badajoz, Spain; 3 Department of Psychology, Universidad Loyola Andalucía, Dos Hermanas, Sevilla, Spain; Rutland Regional Medical Center, UNITED STATES

## Abstract

**Background:**

Khat is a plant that is used for its amphetamine-like stimulant properties. However, although khat is very popular in Eastern Africa, Arabian Peninsula, and the Middle East, there is still a lack of studies researching the possible neurobehavioral impairment derived from khat use.

**Methods:**

A systematic review was conducted to identify studies that assessed the effects of khat use on neurobehavioral functions. MedLine, Scopus, Cochrane, Web of Science and Open Grey literature were searched for relevant publications from inception to December 2020. Search terms included (a) khat and (b) several cognitive domains. References from relevant publications and grey literature were also reviewed to identify additional citations for inclusion.

**Results:**

A total of 142 articles were reviewed, 14 of which met the inclusion criteria (nine human and five rodent studies). Available human studies suggest that long term khat use is associated with significant deficits in several cognitive domains, including learning, motor speed/coordination, set-shifting/response inhibition functions, cognitive flexibility, short term/working memory, and conflict resolution. In addition, rodent studies indicated daily administration of khat extract resulted in dose-related impairments in behavior such as motor hyperactivity and decreased cognition, mainly learning and memory.

**Conclusions:**

The findings presented in this review indicates that long-term khat use may be contributing to an impairment of neurobehavioral functions. However, gaps in literature were detected that future studies could potentially address to better understand the health consequences of khat use.

## Introduction

Khat refers to the shoots and leaves of the plant *Catha Edulis* Forsk, which is endemic in the countries around the Red Sea and Eastern Africa, and that has been used for centuries for its stimulant properties [1,2]. Young shoots and leaves are used to alleviate fatigue, enhance work capacity, stay alert, reduce hunger, and induce euphoria and self-esteem [3,4]. To date, no study has effectively calculated the global prevalence of khat use [5]; it is estimated that approximately 5–20 million people worldwide consume khat [6,7]. Although little is known about the neuroendocrine, neurophysiological, and neurochemical effects of khat use in humans [8], daily khat use has been associated with multiple social, medical and mental health problems, including psychosis, depression and self-harm [2,9–11].

Cathine and cathinone are the main active constituents of khat and, in terms of structure and effects, are comparable to amphetamines [12,13]. Cathinone accounts for most stimulant effects on the Central Nervous System (CNS), increasing concentrations of stimulant neurotransmitters such as dopamine, serotonin and/or noradrenaline in specific brain regions, in the striatum [14–16]. As a result, the structural and pharmacological parallels between khat cathinone and amphetamines offer a model for understanding the long-term effects of khat use, which could be comparable to cognitive deficits such as learning and cognitive flexibility associated with prolonged amphetamine use [17, see 18 for a review]. While empirical research addressing the effects of chronic khat administration are still scarce, there is growing scientific evidence in human and animal studies that demonstrate cognitive deficits. Several studies have shown that khat is an addictive neurotoxic substance with an effect neurobehavioral functions [12,13,19,20]. In a rodent study, both subchronic and chronic exposure to khat extract was found to cause deficits in short-term memory [21]. Similarly, dose-dependent neurobehavioral effects were reported in another study, whereby repeated exposure to doses of khat extract (100-400mg/kg) was observed to enhance locomotor activity and impair cognitive performance [22].

Despite being a developing issue of global health significance, comprehensive reviews and research examining the impact of khat use on executive function are still lacking. To date, there have only been three reviews published on the neurobehavioral consequences of khat [17,23,24]. Prior to the first paper by Hoffman and al’Absi [17], there were no published studies on the effect of khat use on cognition; therefore, this narrative paper provided a brief literature review and directions for future neurobehavioral studies. This review primarily included broader evidence on cognitive deficit from previous studies on stimulant drugs such as amphetamines [25] and methamphetamine [26,27] to understand the potential behavioral and cognitive effects of khat in humans. In another review, Berihu and Asfeha [24] assessed the level of evidence for the impact of khat on neurobehavioral functions. Results from this meta-analysis revealed a significant association between daily khat use and cognitive flexibility, working memory, learning memory, and motor activities. Finally, the most recent systematic reviews and meta-analysis were explicitly focused on the relationship between khat and memory dysfunctions [23]. The pooled results from this review suggested that chronic khat use was associated with a short-term memory discrepancy. However, these earlier meta-analysis reviews [23,24] did not capture all publications on the subject under review in this current paper; specifically, key executive control components: inhibition, updating and shifting [28,29] that have previously been linked to drug addiction [30] In light of current debates concerning the increase of khat use in East Africa [31] and its contributing factor in cognitive deficits development, a new and updated review was warranted.

This systematic review aimed to clarify the relationship between khat, behavioral and cognitive dysfunction by synthesizing studies investigating the effects of khat use on both human and rodents’ neurobehavioral functions. Therefore, this review’s findings could help to better understand (1) the current pieces of evidence on cognitive and behavioral consequences and (2) to identify potential areas to investigate in future khat research.

## Method

PRISMA guidelines for reporting systematic reviews were followed (S1 PRISMA Checklist) [32] and the protocol was registered in PROSPERO on January 17, 2020 (registration No.: CRD 42020159580). Comprehensive literature searches of PubMed Medline, Scopus, Cochrane Database, Web of Science, and Open Grey Repository databases were conducted, from inception to January 19, 2020, and last updated on December 2020. Databases were searched separately by two reviewers (DMR and MJR). The search strategy (see S2 Table) incorporated combinations of two different concepts: (a) khat; and (b) cognitive domains. Searches were piloted in PubMed and then adapted to run across the other databases. To identify any additional articles, the reference lists of the included studies and recent reviews in the field were checked. In addition, expert authors in the field were contacted.

### Eligibility criteria

The rationale for our inclusion criteria was to have an extensive assessment of the relationship between khat use and different cognitive domains. According to previous reviews on khat and models of stimulant drugs administration, neurobehavioral functions included were: inhibition, mental flexibility, working memory, response conflict, problem-solving, memory, visual and verbal abilities, learning, speed of processing, and social cognition [17,33–38].

Based on previous studies investigating khat, we focused on adults (between 18 to 60 years) because they are the majority of khat users [39], as well as rodents, as it is a well-accepted animal model to test neurobehavioral functions [40]. We focused on studies that assessed executive functions in habitual or chronic khat users, as well as khat-administered rodents by means of cognitive or neuropsychological tasks/batteries in case-control studies, including at least a khat-free control group to avoid cofounding factors of studies using non-khat free control groups or other non-controlled settings (e.g. community or educational) where extraneous variables are not controlled.

### Selection of studies

Study selection was done in duplicate (AA and MJR), and a third reviewer participated in cases of disagreement (RP). First, duplicate studies were deleted. Second, based on the screening of the title and abstract a selection of potentially relevant articles was made. Finally, after reading the full text, a final selection was made. The Kappa inter-agreement statistic was moderate (κ: 0.606; 95% CI: 0.297–0.915). The studies included met specific inclusion and exclusion criteria (see Table 1).

**Table 1:** Inclusion and exclusion criteria for the studies included in the review.

Aspects considered	Inclusion criteria	Exclusion criteria
Population	• Habitual khat users • Long-term khat users • Adults • No limit of consumption • Animal studies (rodents)	• Past khat users • Occasional users • Under 18 years-old
Intervention, Exposure	• Adults who use khat, • Quasi-experiments, Lab-based experiments • cross-sectional studies, Longitudinal studies, No limits of addiction • Executive functions tasks /assessment • Neuropsychological batteries • Neuroimaging techniques (fMRI and EEG only)	• Non-cognitive/behavioural task use • Non-neuropsychological testing • Non-neuropsychological tasks • Other community/educational settings • No khat-free control group
Comparator	• Amphetamine • Cathinones (synthetic) • Khat-free controls (adults who do not use khat)	• The study has no comparator other than khat or tobacco
Outcome	• Impaired cognitive / Neurobehavioral processes • EEG • FMRI	• Metabolic processes • Psychological assessment • Only psychosocial assessment (depression, anxiety, personality traits, quality of life…)
Design	• Experimental and quasi-experimental with control groups	• Rest of designs
Language	• All languages	• None
Setting	• Laboratory	• School, community, non-laboratory settings

Note. EEG: Electroencephalogram; fMRI: Functional magnetic resonance imaging.

### Data extraction

A data extraction sheet was developed, pilot tested and refined it accordingly. The main characteristics of these studies were rigorously extracted by AA and verified by a second reviewer (MJR). For each study, information was collected about the authors, year of publication, study country, sample size, age, sex, intervention and comparators, cognitive domain and tasks or batteries, and main results.

### Risk of bias in individual studies

Quality assessment was performed independently in duplicate (DMR and MJR), and a third reviewer participated in cases of disagreement (AA). The quality of animal studies was assessed with the SYRCLE tool [41]. The quality of control case studies was assessed with the Newcastle-Ottawa Scale (NOS) [42]. The SYRCLE tool assesses six categories within ten domains, assigning a judgment of low, high or unclear risk of bias to each domain. The NOS awards stars for three categories: selection, comparability, and exposure. The maximum number of stars that can be achieved in a study with the NOS is nine, which indicates a complete absence of bias.

## Results

### Search results

**Fig 1:**
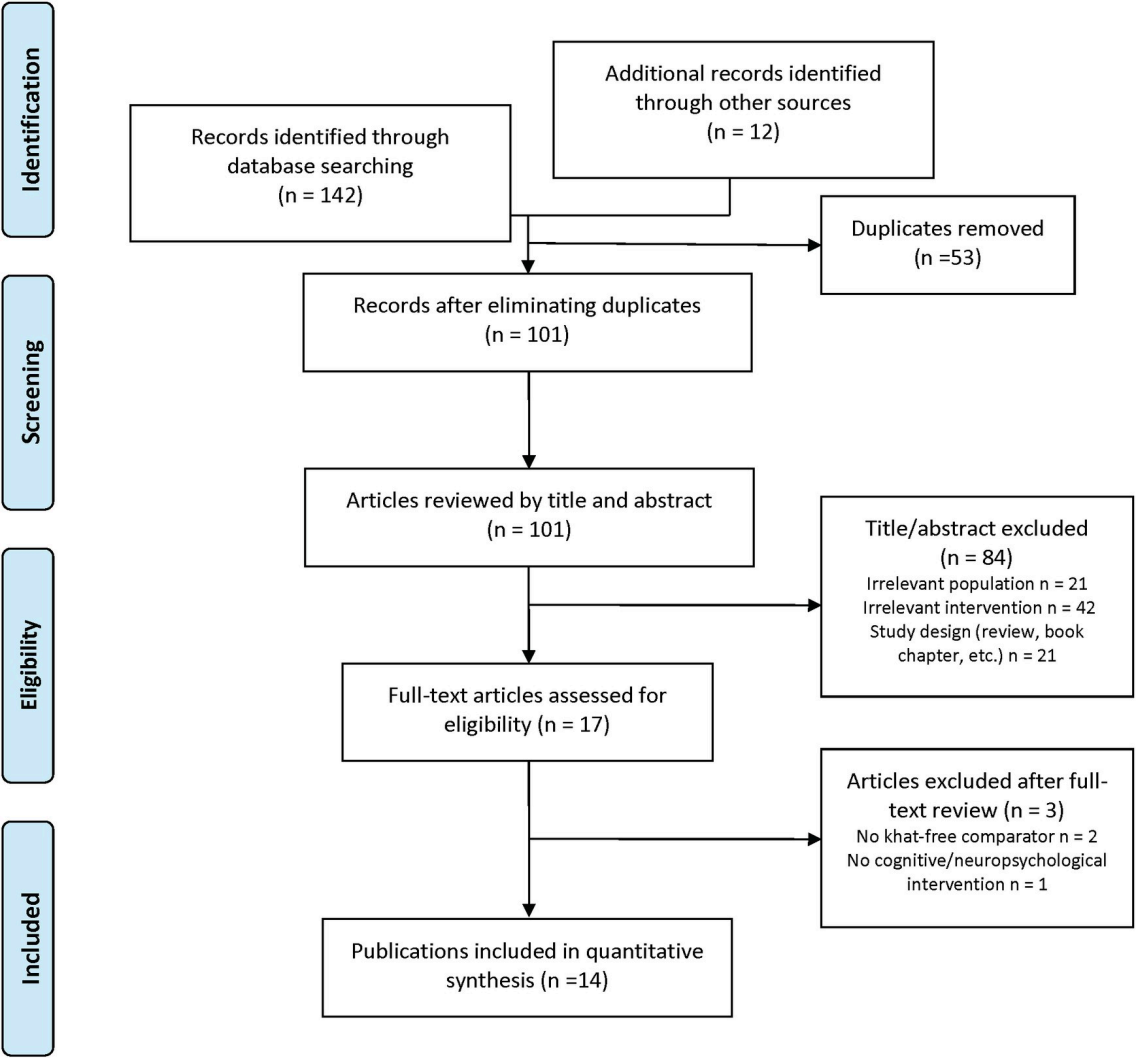
Flow chart of articles included and excluded after the systematic review.

The search strategy produced 142 potentially relevant studies (see Fig 1 PRISMA flow diagram). Further 12 articles were identified from the references of the articles selected. Of these, 53 were duplicates. Of those remaining, 84 were excluded after reviewing the title and abstract. After reviewing the full text of the remaining articles, three was excluded for the following main reasons: (1) did not test a cognitive domain or (2) no appropriate control group. Finally, 14 articles were selected, nine case-control human studies and five rodent studies (including four mice and one rat models) (Tables 2 and 3).

**Table 2:** Description of the human studies included in the present systematic review.

Study, Country study^a^	Design	Sample size (intervention/control)	Mean age (*SD*)	Sex (men/women)	Intervention /comparators	Cognitive domain / Task or battery	Main results	Summary
Colzato, Ruiz, van den Wildenberg, & Hommel [43] (NL)	Cross-sectional	40 (20/ 20)	31.3 (6.5) khat users / 30.7 (5.8) controls	36 / 4	Chronic use / Khat-free controls	Cognitive flexibility and Working Memory Updating / Global Local task and N-Back task	ANOVA • Global-Local task: Khat users Vs. Control Switch effect size, group effect: F[1, 36] = 5.68, *p* = < .05, MSE = 8073.45, *η*^*2*^_*p*_ = .17* • N-Back task: Khat users Vs. Control. Accuracy -1-Back: *t*(36) = 4.72, *p* = .001*** -2-Back: *t*(36) = .75, *p* = .001***	Khat use impairs cognitive flexibility and working memory
Colzato, Ruiz, van den Wildenberg, Bajo, et al. [44] (NL)	Cross-sectional	40 (20/20)	31.3 (6.5) khat users / 30.7 (5.8) controls	36 / 4	Chronic use / Khat-free controls	Inhibitory control / Stop-Signal Paradigm	ANOVA • SSRTs^1^: Khat users Vs. Control: F[1,38] = 33.21, *p* < .001, MSE = 584.624, *η*^*2*^_*p*_ = .47.*** • SSRTs^1^ to Go signals as a covariate: Khat users Vs. Control: F[1,38] = 29.97, *p* < .001, MSE = 599.701, *η*^*2*^_*p*_ = .44.***	Khat use Impairs inhibitory control
Colzato et al. [45] (NL)	Cross-sectional	32 (16/16)	32.4 (7.2) khat users / 30.7 (5.8) controls	28 / 4	Chronic use / Khat-free controls	Interference control / Simon task	ANOVA • Reaction time: Khat users Vs. Control: Reaction time 3-way interaction F[1, 29] = 5.71, *p* < .05, MSE = 165.08, *η*^*2*^_*p*_ = .16*	Chronic khat use impairs response conflict
Colzato et al. [46] (NL)	Cross-sectional	29 (11/18)	31.5 (5.4) khat users / 20.8 (3.0) controls	26 / 3	Acute use / Khat-free controls	Interference control / Simon task	ANOVA • Khat users Vs. Control Correspondence and Group Interaction effect (Second Block: F[1, 27] = 3.89, *p* = .06, MSE = 373.97, *η*^*2*^_*p*_ = .13*	Acute khat exposure could potentially enhance the ability to resolve response conflicts
Hoffman & Al’Absi [47] (Y)	Cross-sectional	58 (32/26)	24.2 (5.3) khat users / 22.9 (4.8) controls	39 / 19	Chronic use / Khat-free controls	Working memory and Speed of information processing / Forward and backward digit span test (FDST and BDST) and Digit Symbol Substitution Test (DDST)	MANCOVA with age and education as covariates • FDST: F[3, 57] = 0.19, *p =* .66^ns^ • BDST: F[3, 57] = 4.511, *p* = .007** • DDST: F[3, 57] = 0.82, *p =* .37^ns^	Khat users scored worse on the digit backward measure of short-term memory.
Hoffman & Al’Absi [48] (Y)	Cross-sectional	175 (48/75/52)	25.2 (5.3) Khat and tobacco users / 23.1 (4.4) khat users / 22.4 (3.7) controls	90 / 85	Only Khat chronic use / Chronic khat and tobacco use / Khat-free controls	Verbal learning and verbal memory recall / Arabic version of the Rey Auditory Verbal Learning Test	MANCOVA with age as a covariate • 3-group (concurrent users of khat and tobacco, khat only users, nonusers) × 2 gender (men, women) - trial 2: F[2,174] = 3.886, *p* = .01** - trial 3: F[2,174] = 3.176, *p* = .02* - trial 4: F[2,174] = 2.752, *p* = .03* - trial 5 or best learning (BL): F[2,174] = 2.336, *p* = .05* - delayed recall (20 minutes): F[2,174] = 2.35, *p* = .05*	Tobacco and khat (con-current) use is linked with deficits in verbal learning and delayed recall
Ismail et al. [49] (SA)	Cross-sectional	147 (72/75)	24.3 (4.8) Khat chewers / 22.9 (5.4) nonchewers	142 / 0	Chronic use / nonchewers controls	Learning, episodic and working memory, motor speed/coordination, attention/information processing speed, sustained attention, set-shifting/response inhibition, and perceptual functions / Computerized Behavioral Assessment and Research System (BARS), Trial Making A and B, Block Design, and Benton Visual Retention.	Multiple Linear Regression Analysis • Serial Digit Learning, *B* = -3.8, *p* = .005* • Finger Tapping task taps of nonpreferred hand, *B* = -1.5, *p* = .02* • Trail Making Test: subtest B, *B* = 23.0, *p* = .049*	Chronic khat use decrease functioning in learning, motor speed/coordination, set-shifting/response inhibition functions.
Khattab & Amer [50] (US)	Cross-sectional	78 (25/29/24)	44.6 (6.5) Regular khat chewers) / 38.1 (6.5) Social khat chewers / 44.3 (6.4) Non-khat-chewers	88 / 0	Regular/social—current users / Non-khat-chewers	Perceptual-visual-memory and decision-speed^2^ / Kit of Factor-Referenced Cognitive Test composed (Hidden figure test, Figure Classification test, Map memory test, Identical picture test)	Group One Way ANOVA: • Hidden Figure Test: F ratio = 52.493, *p* < .0001 *** • Figure Classification Test: F ratio = 52.020, *p* < .0001 *** • Map Memory Test: F ratio = 35.917, *p* < .0001*** • Identical Picture Test: F ratio = 57.795, *p* < .0001***	Khat use impairs memory function
Nakajima et al. [51] (Y)	Cross-sectional	175 (49/52/74)	23.8 (0.7) Khat and tobacco / 24.8 (0.5) Khat only / 23.6 (0.6) controls	90 / 85	Chronic khat and tobacco use / Only Khat chronic use / Khat-free controls	Attention and working memory / Mental arithmetic test	ANOVA 3 group (concurrent users of khat and tobacco, khat-only users, and non-users) X 2 gender (men and women). Group effects: Number of correct responses - Group effect: F[2,169] = 5.74, *p* = .004; follow-up tests: *ps* ≤ .03** • Attempts - Group effect: F[2,169] = 5.40, *p* = .005; follow-up tests *p* = .005** • Accuracy rates - Group effect: F[2,169] = 3.63, *p* = .03; follow-up tests: *p* = .03*	Concurrent group perform significantly worse on attention and memory than khat-only and controls

^a^ NL: The Netherlands; SA: Saudi Arabia; US: United Stated of America; Y: Yemen.

^1^ SSRT Stop-Signal Reaction time.

^2^ Decision speed is defined as the time to complete tasks on which errors are likely to be made [52].

ns: Nonsignificant

*** *p* ≤ .001

** *p* ≤ .01

*p ≤ .05, *ps*.

**Table 3:** Description of the animal studies included in the present systematic review.

Study, Country study^a^	Design	Species	Sample size (experimental/control/others)	Mean age	Sex (male/female)	Conditions (Intervention /comparators)	Cognitive domain / Task or battery	Main results	Summary
Afadly et al. [53] (ET)	Confirmatory experiment	Wistar albino rats	18 (6/6 / 6 MPD^1^)	6–8 weeks old	18 / 0	Acute administration / control and MPD^1^	Spatial learning and memory / RAM^2^	Repeated Measures ANOVA • Trial completion time Main effect for Time: F (19,359) = 34.578, *p* < .0001 khat fed groups had significantly higher time to complete the trials compared with control (*p* < .0001) and MPD (*p* < .0001) groups. • Latency Khat and MPD fed groups had significantly lower latency period (faster at entering the arms) than control groups (all *p*<0.0001). • Index for working memory (Working memory errors) Khat fed group had significantly high working memory (correct and incorrect) errors when compared with MPD (*p* = .037 and *p* = .015, respectively) and controls (for both *p* = .001).	Khat fed rats were more affected in working memory.
Bedada & Engidawork [54] (KN)	Confirmatory experiment	Mice	45 (15/15/15 AMP^3^)	6–8 weeks old	not reported / 75 dams^4^	Acute administration / control and AMP	Learning and recall / Multiple T-mazes and Y-maze	ANOVA • T-maze: Increased latency to reach the goal box and higher number of wrong decisions (*p* < .05, in all phases) seen in the K200 and AMP compared with the control group. • Y-maze: All treated groups displayed poorer performance in the Y-maze (*p* < .05)	Daily administration of khat impairs learning and memory
Geresu et al. [55] (ET)	Confirmatory experiment	Albino mice	36 (6/6/24)	6–8 weeks old	30 / 0	Acute administration/control (vehicle Tween 80) and cannabinoid agonists	Motor activity and working memory / Automated Activity Box, Elevated plus maze, and Y-maze	ANOVA - Index for locomotor activity Khat-only treated mice had significantly higher locomotor activity compared to control mice *(p* < .001). - Index for working memory The number of arm entries in the Y-maze test increased in K300 (khat 300 mg / kg) mice compared with control mice (*p* < .05).	Acute exposure increases locomotor activity but does not affect working memory
Kimani & Nyongesa [56] (KN)	Confirmatory experiment	CBA mice^5^	20 (15/5)	5–6 weeks old	20 / 0	Acute administration / control (saline solution)	Spatial learning and memory / modified Morris water maze	ANOVA and *t*-test • Index of learning Mice treated with moderate and high bw^6^ of khat extract had a higher escape latency compared to controls and mice treated with low bw (*p* < .05) • Index of memory - Mice treated with moderate (120 mg/kg) khat extract: higher swim distance (*p* < .05) compared to the other groups. - Swim speed was significantly suppressed (lower) at 360 mg/kg bw compared with mice treated with lower doses of khat as well as controls (*p* < .05). - Mice treated with high dose of khat spent significantly more time in the target quadrant than that of the adjacent NE right quadrant [*t*(4) = 6.15; *p* < .05]	Daily administration of khat extract has a selective effect on learning and memory that appears to be dose-dependent.
Kimani et al. [57] (KN)	Confirmatory experiment	CBA mice^5^	15 treated with three different types doses of khat (40, 120, 360 mg / kg; n = 5 per group) / 5 controls	5–7 weeks old	20 / 0	Acute administration / control (saline solution)	Spatial acquisition, reversal learning and reference (long-term) memory / modified Morris water maze	Paired *t*-test • Index for learning - Escape Latency Mice treated with low (40 mg/kg) khat dose had higher escape latency (*p* < .05) on day 1 of reversal training compared to the baseline performance. Mice treated with a moderate dose of khat extract had shorter escape latency on day 3 and 4 (*p* < .05). Mice treated with higher khat extract had (*p* < .05) increased escape latency across the reversal learning phase compared to baseline performance and controls (t[3] = 2.57 *p* = .05). - Swim distance Swim distance was longer on reversal training day 1 (*p* = .018) and 2 (*p* = .06) in mice treated with low extract of khat. Swim distance in mice treated with 360 mg/kg bw of khat was longer (*p* = .002) on reversal training day 1 compared to baseline performance. - Swim speed Mice treated with 120 mg/kg bw of khat extract swam significantly slower compared to controls (*p* = .003). • Reference (long-term) memory - Post-acquisition probe trial (day 5) All mice treated groups spent (*p* < .05) more time in the target quadrant than in the other quadrants. - Post reversal probe trial (day 9) Mice treated with 120 mg/kg khat extract spent more time in the new target quadrant than compared to other quadrants (all *p* < .05) Mice treated with 360 mg/kg bw had shorter time spent in the adjacent quadrant (t3 = -5.89 *p* = .010) compared to the controls.	Time and dose-dependent inhibition of both learning and reference memory.

^a^ ET: Ethiopia; KE: Kenya.

^1^ Methylphenidate.

^2^ RAM: Radial Arm Maze.

^3^ Amphetamine.

^4^ Offspring’s sex were not reported.

^5^ Cross of a Bagg albino mice.

^6^ bw: body weight.

### Study quality

The results of the quality assessment of the included studies are presented in S3 and S4 Tables.

The total mean NOS was 5.7 (*SD* = 1.5; range 4–8). Of the nine studies, four provided a case definition adequate, and only one study has representativeness of the cases. The analysis of comparability revealed that five studies controlled for age and other substance consumption. Only in one study, the outcome was measured through a blinded assistant. Finally, all the studies employed the same method of ascertainment for cases and control groups.

The SYRCLE tool was employed in five articles. All of them reported baseline characteristics, other sources of bias and incomplete outcome data. However, none of the studies provides sequence generation or allocation concealment. Only one study reported a blind performance and the random housing of the animals included in the study.

### Human studies

Overall data from 774 subjects (481 users and/or concurrent users; 293 non-users (controls); 31% female) was included to review evidence for khat’s effect on human cognition (see Table 2). The included articles reported that khat use was associated with cognitive impairments in different domains, including attention, cognitive flexibility, conflict resolution, decision-making, information processing speed, inhibitory control, learning, motor speed/coordination, short-term memory/working memory, and visual memory [43–51].

#### Executive function

Regarding executive function, three studies examined changes specifically in response to conflict and inhibitory control associated with acute and chronic khat use [44–46]. Colzato et al. [45] examined whether there was a performance difference between long-term khat users and khat free control on the Simon effect test. The Simon effect test is a behavioral measure of interference/conflict resolution in the face of congruent/incongruent stimulus-response trials. The study revealed that chronic khat users were significantly slower than khat-free controls (48 *vs*. 31ms, *p* < .05) in responding to incongruent stimulus-response trials, suggesting long-term khat use impairs cognitive control and the ability to resolve response conflict. In a later study, Colzato et al. [46], assessing acute khat use, they found that the khat group showed a significantly reduced Simon effect in the second task block, performing better than the controls (38 *vs* 59ms, *p* < .05). In sum, the authors found that chronic khat use was associated with negative effects on interference control. In contrast, acute consumption was related to an enhancement in the ability to inhibit behavioral responses. Moreover, individuals who use khat reported to display deficits in inhibiting and executing responses [44]. Using the stop-signal paradigm, authors found khat users to have significantly longer stop-signal reaction time (236ms) compared with khat-free controls (192ms) (*p* < .001).

#### Motor/Information processing speed and set-shifting (cognitive flexibility)

Khat chronic users evidence difficulties related to motor speed and information processing, as a result, perform poorly on tasks that combine such skills with selecting relevant responses [45], the retrieval of information in short-term/working memory [43] and motor inhibition [44]. Ismail and colleagues [49] examined the effects of khat use on the finger-tapping test’s performance. They found that people who use khat displayed impairment in motor speed and coordination function. On the non-dominant hand, khat using individuals had a fewer number of taps than the control subjects. In the same group of participants, Ismail et al. [48] administered Trail Making B test (TMT-B) and found that chronic khat users exhibited significantly slower completion times on the TMT-B test compared to controls (192.4 *vs* 169.4ms, *p* < .05). In line with this, Colzato, Ruiz, van den Wildenberg, and Hommel [43] reported evidence that khat users showed deficits in a task-switching paradigm that specifically examined set-shifting between mental sets and task (known as cognitive flexibility). Khat users were found to have a significant reaction time difference between repetition and alternate trials than healthy controls. These results indicated khat users took longer (greater switching cost) (87 *vs* 37ms, *p* < .05) to redirect attention to respond to different tasks compared to khat-free controls.

#### Working memory

Our literature search identified three studies that examined the effects of khat use on working memory [43,47,51]. Employing the N-back task, Colzato, Ruiz, van den Wildenberg, and Hommel [43] found that khat users showed deficits in working memory associated with updating information when comparing to khat free control group. This impairment was reflected in error rates because khat users were found to commit significantly more errors in both 1-back and 2-back conditions. However, there was no significant difference in reaction times between the groups. Another study found that the effects of concurrent use of khat and tobacco influences working memory and attention [51]. Concurrent users demonstrated poorer performance on working memory, with lower correct responses on a mental arithmetic test compared with khat-only users and healthy controls. In addition, these concurrent users were found to make fewer attempts on the task to generate correct responses than khat only users and displayed a significantly lower accuracy rate than controls.

Three studies have investigated the impact of khat on verbal learning and memory function [47–49]. Concerning learning, Ismail et al. [49] found that individuals that chewed khat demonstrated deficits associated with learning. Compared to non-users, users were found to have significantly lower scores on the Serial Digit Learning test. In an earlier study targeting learning and memory, Hoffman and al’Absi [48] compared the performance of concurrent khat and tobacco users, khat only users and controls on the Arabic version of the Rey Auditory Verbal Learning Test (RAVLT). Authors found that concurrent users demonstrated significantly greater difficulties in recalling words on trials 2–5 and on delayed recall measures of previously learned words. However, the authors did not find differences between chronic users and controls across all the verbal learning and memory recall measures. Thus, the results suggested that concurrent users had impairments in verbal learning and in the ability to retrieve information (after a short delay) from short-term memory. Consistent with this, Hoffman and al’Absi [47] found that chronic khat users compared to controls performed significantly worse in the backward Digit Span Test, in which a sequence of numbers had to be repeated in the reverse order.

#### Visual memory and decision-making process

Finally, only one study assessed visual memory and decision making in khat users Khattab and Amer [50] reported deficits in perceptual visual memory and decision-making process in a population of regular and social khat users. Authors found a significant difference in all four memory subtests of the Kit of Factor-Referenced Cognitive Tests [58], with both khat groups regular and social khat users scoring lower than controls on all the subtests. Additionally, they found differences among khat users, with regular users scoring lower than social users on all the tests.

#### Effect of frequency, duration of khat use on cognitive function

Colzato et al. [46] found a significant positive correlation between the hours spent chewing khat and the Simon effect. Similarly, duration and frequency of khat use along with tobacco consumption and nicotine dependence were found to be important modulators of cognitive performance [50,51]. In addition, it has been suggested that consumption over a longer period could potentially be long-lasting and deleterious to cognitive functions [43,44,51].

### Rodent studies

Overall data from 163 rodents were included to review evidence for the effect of khat on behavior (see Table 3).

#### Effect of khat use on motor behavior

One study explored the effect of daily khat administration on locomotor activity in mice [55]. Authors found that mice treated with khat extract showed significantly enhanced exploration activity compared with the controls, which suggests acute khat exposure alters motor activity in rodents.

#### Learning and memory

All five studies included in the present systematic review have examined the effects of acute khat administration on learning and memory [53–57]. Kimani and Nyongesa [56] found that mice treated with moderate and high khat extract took significantly longer to locate the escape platform than mice treated with low khat extract and controls. In addition, a high dose of khat extract (360mg/kg bw) was found to improve memory performance function significantly, while moderate and low doses impaired accuracy for spatial memory of the platform location.

However, in another study [54] the authors observed increments in latencies and errors (on days 2 and 14) in mice treated with high khat dosage (200 mg/kg bw, daily), which suggests pronounced impairments in both learning and memory. In a second experiment in the same study, all treatment groups had a significantly higher frequency of repeated arms entries compared with the control group in the Y-maze test. Similarly, Geresu et al. [55] found a pronounced spatial working memory deficit in mice treated with higher khat doses (300 mg/kg bw, daily). However, both studies reported that khat treated mice had a lower percentage of alterations in the pattern of arm visits compared to controls, thus exhibiting poorer reward-seeking behavior [54,55].

In rats, Alfadly et al. [53] found that khat increased the speed on entering the arms of the Radial Arm Maze. Specifically, khat fed rat groups were significantly slower in completing the experiment compared to both the control and MPD (methylphenidate) groups. With respect to working memory, authors reported khat fed rats (500 mg/kg bw) were more likely to commit working memory correct errors by repeated entries to a baited arm that no longer had food; and working memory incorrect errors, as evidenced by the animal’s tendency to re-enter the same unbaited arm when compared with MPD (3 mg/kg bw, daily) and control [53].

Kimani and Nyongesa [56] reported that administration of khat has differential dose effects on learning and memory performance in the Morris Water Maze test. This study found that khat administration reduced the swim speed of mice treated with moderate and high doses during the baseline (acquisition) phase compared with controls. After the removal of the escape platform from the maze (day 5), khat exposure was found to not interfere with long-term (reference) memory. However, performance during the reversal learning phase, showed dose-dependent impairments in learning, as indicated by escape latency and swim distance. For instance, mice treated with low (40 mg/kg bw, daily) khat extract displayed longer escape latency (during the first 2 days) and significantly longer swim distance (in day 2) compared to baseline performances. In contrast, high dose (360 mg/kg bw, daily) of khat significantly increased escape latency across the reversal learning trials than baseline performance and control animals. During the post reversal learning probe trial (day 9), mice treated with low and high doses of khat displayed a stronger bias for the former target quadrant compared to the new target, whereas mice treated with moderate khat dose were successful in switching their behavior to learn the new location.

## Discussion

The objective of this systematic review was to provide an updated review of the effects of khat on neurobehavioral performance in both humans and animals. While this review’s conclusions and that of Berihu et al. [23] on the effects of khat use on learning and memory are similar, this paper has identified additional studies not included in the previous reviews. In line with other psychostimulants (cocaine and amphetamine) drugs studies [59,60] acute administration of khat has been observed in one study [46] to improve conflict resolution performance in humans (e.g. resolving stimulus-induced response conflict) as a result of the potential natural effect of khat use [13]. Furthermore, long-term khat use was reported to induce significant deficits in several cognitive domains: learning, motor speed/coordination, short-term/working memory, conflict resolution, decision-making, and visual memory [43,44,47–51]. Specifically, this review contains publications on key executive control domains associated with long-term psychostimulant drugs such as set-shifting/response inhibition functions and cognitive flexibility [43–51]. These findings support previous studies which have demonstrated long-term use of psychostimulant drugs such as cocaine, methamphetamine and amphetamine are associated with detrimental effects on executive functions [27,59,61–63]. Chronic exposure to amphetamine and methamphetamine has been broadly implicated in producing alterations at the neuromodulatory and cortical level [62–65]. Given the chemical resemblance between cathinone and amphetamine, khat is classified as a psychostimulant that could potentially disrupt the dopaminergic pathways within the frontostriatal and limbic regions of the brain, which are responsible for executive (higher-order) functions implicated in the control of addiction [63,66].

Additionally, khat’s neurobiological and behavioral consequences are further complicated with the interactions of other substances such as alcohol and smoking cigarettes (nicotine) [67]. Several studies have reported that many khat users also smoked cigarettes [20,51,68] and that these concurrent users display impairments in verbal learning, memory recall and working memory [48,51]. One explanation is that cathinone has been found to act as a presynaptic release and uptake inhibitor of dopamine [69] leading to depletion of serotonin [70] in brain areas involved in spatial learning and memory [71]. Previous literature suggests an association between nicotine and cognitive deficits in learning and memory [72]. Despite this, it remains unclear the extent to which the observed deficits preceded substance use arises because of concurrent use or might reflect the effect of either khat or nicotine alone. Moreover, it has been suggested that factors such as time spent chewing khat, duration of use, amount (dosage), frequency of use are related to the general slowing degree of impairment in cognition [45,47,48,50,51].

In animals, daily administration of khat extract resulted in dose-related impairments in behavior such as motor hyperactivity and decreased spatial learning, memory as reward-seeking behavior [53,55]. It has been reported that changes such as the escalated behavioral and motor-stimulant responses associated with the repeated daily administration of khat in rodents are typical of behavioral sensitization [53]. Repeated exposure to khat sensitizes stimulant effects and leads to pairing khat to environmental cues that elicit conditioned activity.

Findings from animal studies suggest that khat dosage is the primary determinant of the neurobehavioral effects observed in rodents [53]. Converging evidence from methamphetamine and amphetamine studies reported repeated dosing enhanced behavioral abnormalities responses in a dose-dependent manner [73,74]. Similarly, khat was found to selectively enhance spatial learning and impair working memory [53,56,57]. These differential patterns of learning and memory deficits suggest a loss of cognitive flexibility related to differences in dose and time [57]. More recent findings reported khat extract administered on acute and subacute induced short-term memory discrepancy but had no effect on long-term memory across the treatment regimens [21,75].

### Strengths and limitations

A major strength of this systematic review is that the synthesis of the available literature provided insight into cognitive deficits that were not examined in previous reviews [17,23,24]. However, the studies identified in this review have several limitations that should be addressed in future research. One of the drawbacks of this review is that it is focused on a comprehensive literature search that provides an outline of current findings. Unlike a meta-analysis, this review does not provide statistical estimates of absolute effects nor report causation and effect but rather indicates possible associations between khat use and cognitive impairments.

Another limitation is that most of the studies in this paper have limited generalizability as they are based on relatively small sample sizes and have mainly studied male subjects. In addition, some of these previous studies have failed to account for the role of current or past use of other substances (e.g. nicotine, polysubstance abuse) on cognition. It is not unusual to recruit khat users who use other substance such as alcohol, nicotine, cannabis, and benzodiazepines [76–79]. Additionally, the variability in the included studies’ experimental design and the lack of screening concerning the duration between khat consumption and cognitive testing make it difficult to ascertain more robust conclusions. For instance, only a subset of eligibility criteria appears to have been applied in human studies. The extent to which individuals with premorbid diagnosis such as Attention Deficit Hyperactive Disorder (ADHD) and/or polysubstance users were effectively prescreened and excluded remains uncertain. Thus, making it difficult to conclude if the reported dysfunctions in cognitive performance are exclusively due to khat exposure. For this reason, the observed neuropsychological impairment reported in khat users could potentially be a result of premorbid diagnosis or multiple substances consumption.

Moreover, most rodent studies were not blinded and increased the risk of performance and detection biases. Such biases could result in responding to the treatment group differently, potentially influencing the interpretation and accuracy of the result. Fourth, most of the studies included were conducted in countries where khat is imported, thus limiting the interpretation of our results. It is well documented that khat’s potency and subsequently neurocognitive and behavioral consequences heavily depend on the type, quality, and freshness that drastically decreased after harvesting [80,81]. Therefore, evidence could evolve as more studies are conducted in geographic regions where higher potent khat is readily available. A key issue about using many neuropsychological measures has small or non-representative normative data for non-western populations. Normative “cut-offs” provide fundamental information on what is a normal range to highlight any deficits or disease. Test performance can be influenced by many factors such as culture; therefore, having normative data adjusted to cognitive performance improves the objectivity of the measures. In addition, language and cultural biases can lead to mislabeling differences in cognitive function between users and controls as ‘impairments’ or ‘deficit’, and so further research is needed to confirm these findings.

Although animal models provide crucial information on a drug’s addictive properties, animal studies remain scarce and challenging to replicate in humans because of psychokinetic differences and the varying route and dosage administration. Therefore, it is harder to establish consistency to determine the translational value of these findings to human subjects with different environmental and social factors that could also influence the degree of neurobehavioral impairment [82]. Finally, these selected studies were restricted to laboratory settings and so lack ecological validity, which means the study’s conclusion must be interpreted with care.

### Future studies

To overcome the limitations mentioned above and improve on the quality of both human and animal studies, several recommendations for future research are proposed. First, specific examinations of multiple factors that influence khat use outcomes are needed. That is, studies that looks at genetic, neurobiological, behavioral, environmental, social, cultural, sex differences, psychological factors (mental health), their interactions, and mediating characteristics related to khat use and their relationship with neurobehavioral functions. Other areas of investigation for future research include studies that examine the relationship between varying patterns of khat use and sociodemographic characteristics (e.g. age, education) on cognition and whether abstinence influences cognitive recovery. Specifically, cognitive domains that have been previously identified in amphetamine and methamphetamine studies addressing current neuropsychological gaps such as social cognition, visuoconstruction, and episodic memory that have yet to be fully understood in khat users is recommended. A more specific pre-screening of research participants will be needed in future clinical and rodent preclinical khat studies to account for confounding variables such as sample size, polydrug/concurrent use, premorbid diagnosis (e.g. ADHD) and sex. To improve validity and reliability of any future studies by implementing random allocation and good blinding in RCT studies and quality checklists to reduce potential biases related to performance, selection and detection. Also, incorporating assertion measure such as biochemical tests to verify drug use status and more structured methods to assess the history of mood and psychiatric disorders. Future animal studies need to devise more ecologically relevant models assessing cognition in habitual and social users with varying exposure and doses of khat. Overall, the findings highlight the need to develop further neuropsychological measures for studying substance misuse in diverse populations. Although one neuroimaging study was identified in this review, based on the analysis, it was concluded that the electroencephalogram (EEG) might not have been sensitive enough to detect cases of khat use. Lastly, more empirical neuroscientific studies aimed at improving our understanding of the neural correlates of khat are needed to inform prevention strategies and identify potential risk markers to shape clinical interventions [83].

## Conclusions

The current systematic review has updated previous reviews on the impact of khat use on neurobehavioral functions implicated on everyday tasks’ performance. The findings suggest that khat is associated with deficits in a wide range of cognitive domains, mainly working memory, learning, motor speed/coordination, and set-shifting/response inhibition functions. Prospective studies and randomized control trials are required to determine the underlying neural mechanisms and the interrelationships between khat use and neuropsychological performances. Also, longitudinal multifactor studies on behavioral, environmental, mental and physical health consequences are needed to improve understanding of the long-term consequences of khat use.

## Supporting information

S1 Table PRISMA checklist. (DOC)

S2 Table Search strategy piloted for PubMed. (DOC)

S3 Table Risk of bias in the studies reviewed assess with the NOS-scale. (DOC)

S4 Table Risk of bias in the studies reviewed assess with the SYRCLE’s tool. (DOC)
