# Supplementary material for: Khat and neurobehavioral functions: A systematic review
Source: PLoS One. 2021 Jun 10;16(6):e0252900. doi: 10.1371/journal.pone.0252900 (PMC8192015; doi:10.1371/journal.pone.0252900)
Supplement: S2 Table — (DOC) [file pone.0252900.s002.doc]

**S2 Table. Search strategy piloted for PubMed**

| **Query** | **Field** | **Search term** |
| --- | --- | --- |
| #1 | Title/Abstract | khat OR qat OR "catha edulis" OR miraa |
| #2 | Title/Abstract | cognitive attention" OR "inhibitory control" OR inhibition OR "working memory" OR "visual memory" OR "visual learning" OR "verbal learning" OR "verbal memory" OR "verbal fluency" OR "verbal fluency" OR language OR "speed of processing" OR "social cognition" OR visuo-spatial OR impulsiv* OR "executive function" OR "problem solving" OR neurobehavioral OR neurobehavioural OR "cognitive control" OR "response conflict" |
| #3 | N/A | #1 AND #2 |
