# Supplementary material for: Khat and neurobehavioral functions: A systematic review
Source: PLoS One. 2021 Jun 10;16(6):e0252900. doi: 10.1371/journal.pone.0252900 (PMC8192015; doi:10.1371/journal.pone.0252900)
Supplement: S3 Table — (DOC) [file pone.0252900.s003.doc]

**S3 Table. Risk of bias in the studies reviewed assess with the NOS-scale**

| **Study** | **Selection** | | | | **Comparability** | | **Exposure** | | |  | |
| --- | --- | --- | --- | --- | --- | --- | --- | --- | --- | --- | --- |
|  | Is the case definition adequate? | Representativeness of the cases | Selection of controls | Definition of controls | | Comparability of cases and controls on the basis of the design or analysis (control for age and other consumption) | Ascertainment of exposure | Same method of ascertainment for cases and controls | Non-response rate | | Assessment of risk of bias NOS |
|  |  |  |  |  | |  |  |  |  | |  |
| Colzato, Ruiz, van den Wildenberg, & Hommel [47] | * |  | * | * | | */* |  | * | * | | 7 |
| Colzato, Ruiz, van den Wildenberg, Bajo, et al. [48] |  |  | * | * | | */* |  | * | * | | 6 |
| Colzato et al. [45] | * |  | * | * | | */* |  | * | * | | 7 |
| Colzato et al. [46] | * |  | * | * | | */* | * | * | * | | 8 |
| Hoffman & al’Absi [49] |  |  | * | * | | */* |  | * | * | | 6 |
| Hoffman & al’Absi [50] |  |  | * | * | | / |  | * | * | | 4 |
| Khattab & Amer, [53] | * | * | * |  | | / |  | * | * | | 5 |
| Ismail et al. [51] |  |  | * | * | | / |  | * | * | | 4 |
| Nakajima et al. [52] |  |  | * | * | | / |  | * | * | | 4 |
|  |  |  |  |  | |  |  |  |  | |  |
|  |  |  |  |  | |  |  |  |  | |  |
