# Supplementary material for: Khat and neurobehavioral functions: A systematic review
Source: PLoS One. 2021 Jun 10;16(6):e0252900. doi: 10.1371/journal.pone.0252900 (PMC8192015; doi:10.1371/journal.pone.0252900)
Supplement: S4 Table — (DOC) [file pone.0252900.s004.doc]

**S4 Table. Risk of bias in the studies reviewed assess with the SYRCLE’s tool**

| **Study** | **Selection bias** | | | **Performance bias** | | **Detection bias** | | **Attrition bias** | **Reporting bias** | **Other** |
| --- | --- | --- | --- | --- | --- | --- | --- | --- | --- | --- |
|  | Sequence generation | Baseline characteristics | Allocation concealment | Random housing | Blinding | Random outcome assessment | Blinding | Incomplete outcome data | Selective outcome reporting | Other sources of bias |
| Alfadly et al. [56] | No | Yes | No | No | No | Not reported | No | Yes | No | yes |
| Bedada & Engidawork [57] | No | Yes | No | Yes | Yes | Not reported | Yes | Yes | No | Yes |
| Geresu et al. [55] | No | Yes | No | Not reported | No | Yes | No | Yes | no | Yes |
| Kimani & Nyongesa [59] | No | Yes | No | No | Not reported | Yes | No | Yes | No | Yes |
| Kimani et al., [58] | No | Yes | No | No | Not reported | Not reported | No | Yes | No | Yes |
